# A method for assessing tissue respiration in anatomically defined brain regions

**DOI:** 10.1038/s41598-020-69867-2

**Published:** 2020-08-06

**Authors:** Erica Underwood, John B. Redell, Jing Zhao, Anthony N. Moore, Pramod K. Dash

**Affiliations:** Department of Neurobiology and Anatomy, The University of Texas McGovern Medical School, Houston, TX 77030 USA

**Keywords:** Biological techniques, Neuroscience

## Abstract

The survival and function of brain cells requires uninterrupted ATP synthesis. Different brain structures subserve distinct neurological functions, and therefore have different energy production/consumption requirements. Typically, mitochondrial function is assessed following their isolation from relatively large amounts of starting tissue, making it difficult to ascertain energy production/failure in small anatomical locations. In order to overcome this limitation, we have developed and optimized a method to measure mitochondrial function in brain tissue biopsy punches excised from anatomically defined brain structures, including white matter tracts. We describe the procedures for maintaining tissue viability prior to performing the biopsy punches, as well as provide guidance for optimizing punch size and the drug doses needed to assess various aspects of mitochondrial respiration. We demonstrate that our method can be used to measure mitochondrial respiration in anatomically defined subfields within the rat hippocampus. Using this method, we present experimental results which show that a mild traumatic brain injury (mTBI, often referred to as concussion) causes differential mitochondrial responses within these hippocampal subfields and the corpus callosum, novel findings that would have been difficult to obtain using traditional mitochondrial isolation methods. Our method is easy to implement and will be of interest to researchers working in the field of brain bioenergetics and brain diseases.

## Introduction

Mitochondria form a complex, interconnected network within cells that continually changes and adapts to meet the energy demands of the various cellular compartments^1–3^. In addition to generating ATP, mitochondria are involved in sequestering calcium, generating reactive oxygen species (ROS), and triggering apoptotic cell death^4–6^. Unlike other organs, the brain does not store excess energy, and its normal functioning is critically dependent on the continuous synthesis of ATP. Altered mitochondrial function has been reported to contribute to brain pathology after stroke and traumatic brain injury (TBI), and has been linked to numerous neurodegenerative diseases^7–19^. Importantly, many neurodegenerative disease pathologies have been shown to originate in specific brain regions (e.g. entorhinal cortex for Alzheimer’s disease), before progressing over time to other brain areas^20–25^. Thus, it would be informative to examine mitochondrial respiration within discrete brain regions to examine its relationship to evolving pathology.

Traditionally, mitochondrial respiration is assessed following isolation from bulk tissue. This technique has a number of disadvantages, including mechanical disruption of the mitochondrial network, and assaying activity in the absence of the mitochondria’s native intracellular environment^26–28^. Furthermore, this method does not lend itself to high resolution spatial mapping of mitochondrial function due to the relatively large amount of input tissue required for mitochondrial isolation. Although cultured neurons can be used to examine mitochondrial respiration in a cellular context, in vitro cultures do not recapitulate the complex three dimensional brain anatomy and heterogeneous cellular environment that exists within the brain, especially in experimental models of brain diseases. Seahorse XF analyzers (marketed by Agilent) are being used to assess mitochondrial function by measuring their oxygen consumption rate (OCR) in response to modulators of key components of the electron transport chain. While these instruments have been used to assess mitochondrial function in intact tissues^29–33^, it has proven to be challenging to measure brain tissue respiration in anatomically defined regions in a high-throughput manner.

Here, we describe a method to measure mitochondrial OCR using small biopsy punches taken from discrete brain regions. The method includes a procedure for maintaining brain tissue viability prior to excising punches, and outlines considerations for determining optimal punch size, and adequate OCR and drug responses. We demonstrate that our method can be used to measure mitochondrial respiration in well-defined anatomical subfields of the hippocampus, as well as in white matter tracts. Finally, we employed our method to show that mild traumatic brain injury (mTBI) results in distinct mitochondrial responses within different hippocampal subfields, a finding which has not been described previously. This method is easily adaptable to meet specific experimental requirements, and can be used to map changes in mitochondrial function in different brain regions/subregions that may arise from many different pathological conditions or disease states.

## Results

### Brain mitochondrial respiration

**Figure 1:**
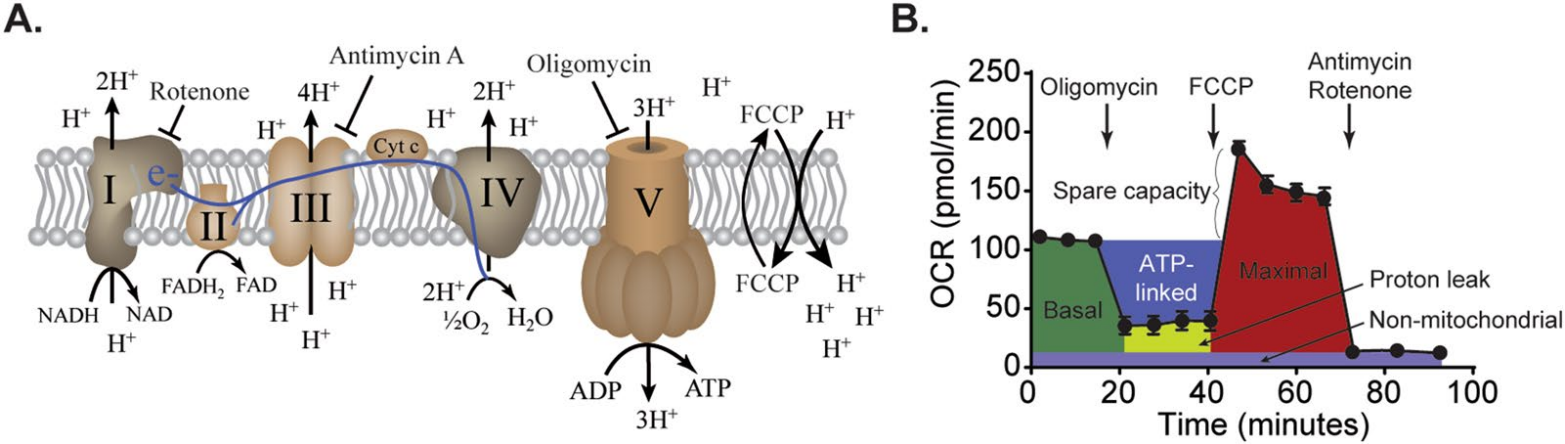
Oxygen consumption rate (OCR) measurements of purified mitochondria. (**A**) Simplified drawing of the electron transport chain located within the inner mitochondrial membrane. Electron transfer is coupled to the transfer of protons (H^+^) across the inner mitochondrial membrane into the inner membrane space, creating a proton gradient. This gradient is utilized by complex V for ATP synthesis. The protons react with oxygen to generate water. Thus, the OCR can be monitored by the Seahorse XF analyzers and used as a surrogate of mitochondrial respiration. The targets of the inhibitors (oligomycin, antimycin A, and rotenone) and uncoupler (FCCP) are indicated. (**B**) A representative OCR curve generated using isolated mitochondria showing the characteristic responses to mitochondrial inhibitors and the uncoupler FCCP.

In the Seahorse metabolic analyzer, mitochondrial function can be assessed by real-time monitoring of the oxygen consumption rate (OCR; primarily consumed by glucose metabolism through the citric acid cycle). Figure 1A shows a stylized drawing of the mitochondrial respiratory chain indicating the sites of action for the drugs and uncouplers that are commonly used to manipulate different components of mitochondrial respiration. Figure 1B shows the impact of these compounds on an OCR curve obtained using isolated rat cortical mitochondria. Inhibitors that target specific respiratory complexes are used to dissect the contribution of the different respiratory chain complexes toward basal respiration, ATP-linked respiration, proton leak, and maximal respiration (basal respiration + spare capacity). Basal mitochondrial respiration is determined by monitoring OCR in the absence of any inhibitors. In isolated mitochondria, the basal respiratory measurements (obtained without ADP) are referred to as state II respiration. The complex V (F1–Fo ATP synthase) inhibitor oligomycin decreases proton flux through complex V, thereby causing the accumulation of protons within cristae, which results in a reduction of electron transport and oxygen consumption. The amount of reduction in OCR represents, in intact cells, ATP synthesis-linked respiration, or in isolated mitochondria, state IV respiration. The mitochondrial OCR remaining after oligomycin treatment is considered to be a measure of proton leak. Carbonyl cyanide-4 (trifluoromethoxy) phenylhydrazone (FCCP) is an uncoupling agent that collapses the proton gradient and disrupts the mitochondrial membrane potential. This results in uninhibited electron flow through the electron transport chain and maximal oxygen consumption by complex IV. The difference between maximal and basal OCR is considered to be the spare (or reserve) capacity. Finally, all mitochondrial-associated respiration is abolished by the addition of rotenone (a complex I inhibitor) and antimycin A (an inhibitor of cytochrome C reductase). The residual OCR observed after addition of these two compounds is carried out by non-mitochondrial oxygen-consuming enzymes that may be present in the sample. The non-mitochondrial fraction is subtracted from all other values to ensure that only mitochondrial respiration is assessed.

Respiration assays using purified mitochondria in Seahorse XF analyzers have been performed by a number of investigators^34–36^. In order to address if the different phases of respiration observed using biopsy size tissue brain tissue punches are qualitatively similar to the phases obtained using isolated mitochondria, we first obtained OCR curves from isolated mitochondria (1 µg/well) using the Seahorse XFe96 analyzer. Figure 1B shows the different states of respiration obtained using mitochondria isolated from ~ 100 mg of cortical tissue and the respiration inhibitors indicated in Fig. 1A.

### Preparation and temporary storage of brain tissue sections

**Figure 2:**
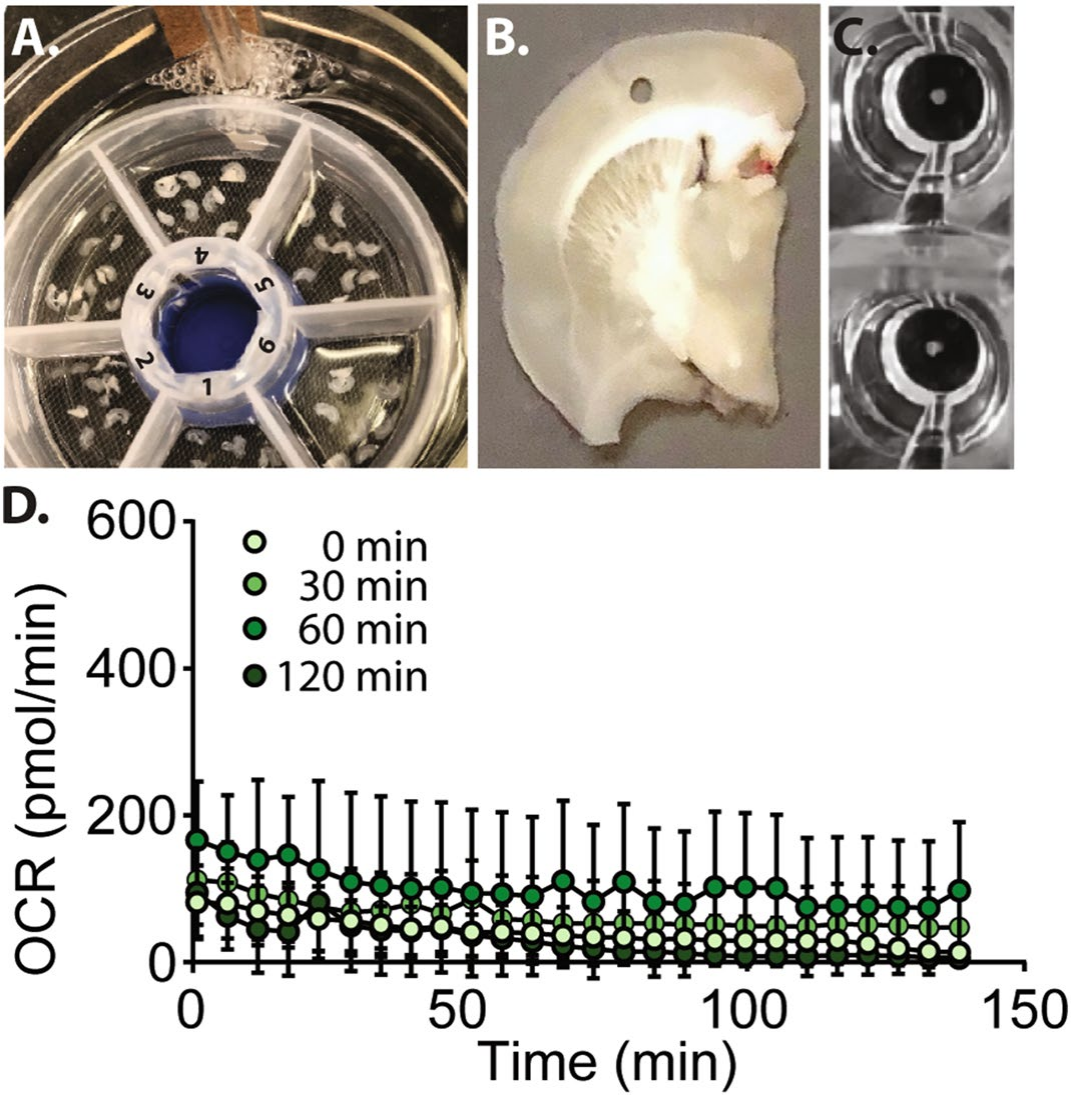
Incubation of rat brain sections for various lengths of time prior to preparing tissue punches does not influence basal OCR. (**A**) Picture of the multi-segment storage chamber designed for maintaining tissues in oxygenated artificial cerebrospinal fluid. Each chamber contains brain sections from a separate animal. (**B**) Picture of a representative tissue section showing the location of the punches used to assess tissue viability. (**C**) Tissue punches need to be centered in the well prior to starting the assay to ensure accurate OCR measures. (**D**) Summary data showing basal respiration, monitored over time, in tissue sections rested for various lengths of time prior to the preparation of cortical punches. Data are presented as mean ± SEM.

As brain sections are highly vulnerable to oxygen and glucose deprivation, we adapted a method commonly used in slice electrophysiology to maintain tissue viability^37–39^. Coronal brain sections (220 µm in thickness) are prepared using the tissue chopper, and sections containing the regions of interest placed in oxygenated artificial cerebrospinal fluid (aCSF) prior to preparing the biopsy punches (Fig. 2A). Since brain tissues from multiple animals would be used in a typical experiment design, we questioned if maintaining the brain sections in oxygenated aCSF would influence tissue viability and mitochondrial respiration. To examine this, rats were euthanized and the prepared brain slices incubated at room temperature in oxygenated aCSF for either 0 min, 30 min, 60 min or 120 min prior to excising 0.75 mm diameter cortical tissue punches for measuring basal respiration. Figure 2B shows a representative tissue slice indicating the site of the cortical biopsy punches used in this experiment. Tissue punches were placed in the center of each XFe96 Extracellular Flux Assay sensor cartridge well (Fig. 2C). This step is critical to obtain reproducible OCR measurements. As the injection of the various mitochondrial inhibitors (and subsequent mixing) can result in punch movement, the position needs to be checked after the completion of the assay. The results from any punch found to have moved during the procedure should be removed from the final analysis. The results shown in Fig. 2D indicate that a stable initial OCR was observed between tissue punches taken from brain sections incubated in aCSF from 0 to 120 min. Furthermore, there was no significant difference in basal OCR measured over the 2 h monitoring period between punches taken at different times.

### Relationship between punch diameter and basal OCR

**Figure 3:**
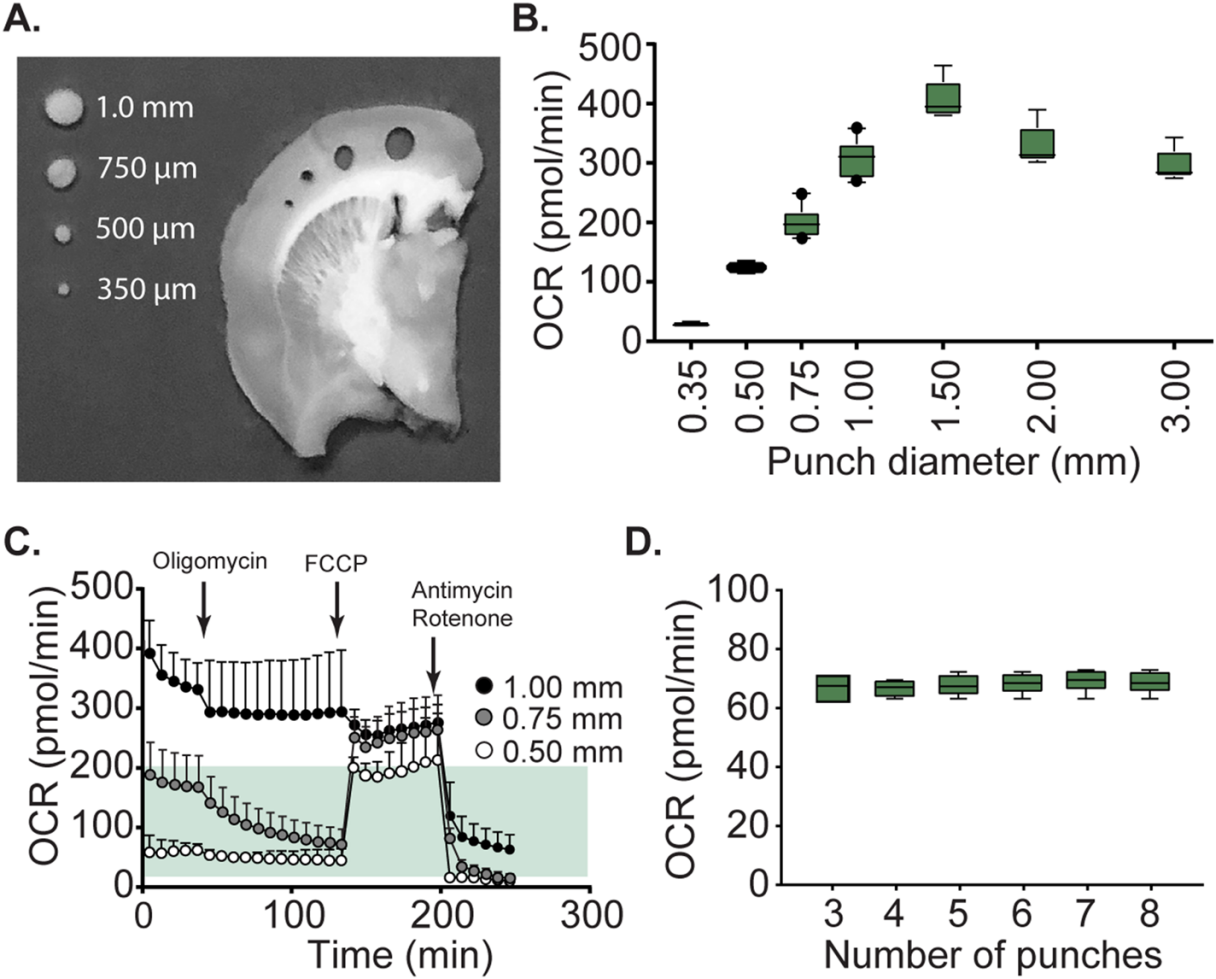
Relationship between basal respiration and cortical punch diameter. (**A**) Picture of a rat coronal tissue section (220 µm in thickness) showing the relative sizes of the cortical tissue punches excised to determine optimal punch diameter for OCR measures to be within the recommended range (20–200 pmol/min). Please note, for evaluation of appropriate punch size, punches were taken from the same anatomical location across brain sections and across animals. (**B**) Box (25th and 75th percentile) and whisker (5th and 95th percentile) plots showing the basal OCR for the different sizes of punches. (**C**) OCR curves showing the response of various punch diameters to mitochondrial inhibitors/FCCP. Recommended range for OCR is indicated by the shaded area. Data are presented as mean ±   SEM. (**D**) Box (25th and 75th percentile) and whisker (5th and 95th percentile) plots showing the relationship between number of replicates and recorded basal OCR for 0.5 mm diameter cortical tissue punches.

The manufacturer of the Seahorse XFe96 recommends that basal OCR should fall be between 20–200 pmol/min in order to obtain reliable OCR measurements, and to minimize potential floor and ceiling effects in response to drug administration. Therefore, we measured the relationship between basal OCR and cortical tissue punch diameter (Fig. 3A) in order to determine the optimal punch size to yield a baseline OCR value within this recommended range. Cortical tissue punches from 220 µm thick coronal sections were used for these studies. Figure 3B shows that punch diameters ranging from 350 µm to 1.5 mm yielded basal respiration values that increased in proportion to punch diameter. However, in punches larger than 1.5 mm diameter, the basal OCR decreased, possibly due to substrate exhaustion and/or oxygen depletion. Furthermore, Fig. 3C shows that while punch diameters of 500 µm remained in the optimal zone for OCR measurement (shaded area) and responded to FCCP, the 1 mm diameter punches failed to do so. Although cortical punch diameters between 350 µm and 750 µm were found to have basal OCR values between the recommended range of 20–200 pmol/min, the OCR in response to drugs (i.e. oligomycin and FCCP) were found to be outside this range and are therefore not recommended for use. Similar experiments carried out using hippocampal punches centered on the CA1 subfield revealed that punch diameters from 500 to 750 µm were appropriate for OCR measurements (Supplemental Fig. 1), illustrating the need for optimization of tissue punch diameter for each region to be assessed. Using 500 µm cortical tissue punches, we assessed the relationship between number of replicates and variability of the OCR measurements^40^. Cortical tissue punches (approximate location shown in Fig. 2) from same location from adjacent brain slices were excised, and placed in individual wells. Basal respiration was measured for each punch and the coefficient of variability (CV = S.D./mean) was calculated by increasing the number of replicate punches included in the group. We determined that 4 punches/structure/animal would effectively reduce the CV to ≤ 5%. The inclusion of additional replicates did not dramatically reduce CV nor did it significantly alter the mean OCR (F = 0.0628, p = 0.992; Fig. 3D).

### Optimization of mitochondrial inhibitors

**Figure 4:**
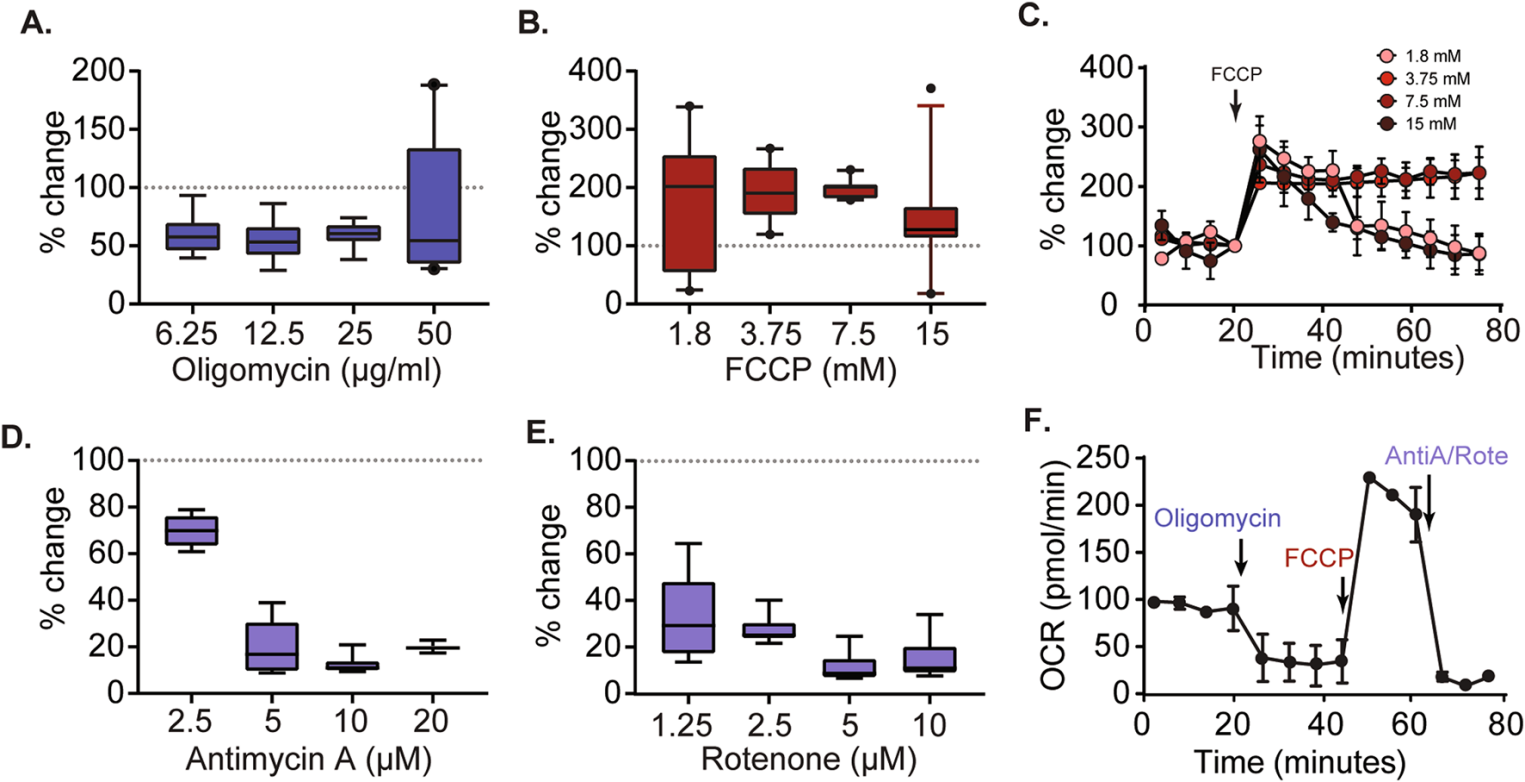
Optimization of respiration inhibitor and FCCP concentrations. Box and whisker plots (generated using 0.5 mm diameter cortical tissue punches) demonstrating the maximum responses to increasing concentrations of (**A**) the ATP synthase inhibitor oligomycin, (**B**) the uncoupler FCCP, (**D**) the complex III/ cytochome C inhibitor antimycin A, and (**E**) the complex I inhibitor rotenone. (**C**) Representative OCR curves showing the responses over time of 0.5 mm cortical tissue punches to increasing concentrations of FCCP. (**F**) Optimal doses of each agent (25 µM oligomycin, 7.5 µM FCCP, 5 µM antimycin A, and 10 µM rotenone) were used to generate a characteristic OCR curve in rat cortical punches.

As stated above, the Seahorse XF analyzers can assess different aspects of mitochondrial respiration by adding specific mitochondrial complex inhibitors and uncoupling agents. Under some circumstances, tissue respiration may cause substrate/oxygen depletion over time. Therefore, we aimed to maintain the total assay reaction time to under 2 h^41^. In order to accomplish this, we tested increasing concentrations of the mitochondrial inhibitors to measure the degree of inhibition and the length of time required to obtain stable OCR readings. Figure 4A shows the percent change in OCR (relative to baseline) of cortical tissue punches (220 µm thick, 0.5 mm diameter) treated with various concentrations of oligomycin. Oligomycin treatment greater than 25 µg/ml showed high OCR variance between replicate samples, while doses from 6.25 to 25 µg/ml were found to effectively decrease OCR, with 25 µg/ml demonstrating the lowest variance across replicates. When similar experiments were carried out using the metabolic uncoupler FCCP (co-injected with 7.5 mM pyruvate to provide additional substrate to meet the higher OCR resulting from uncoupling of the proton gradient and ATP synthesis), an increase in OCR was recorded for doses ranging from 1.875 µM to 15 µM (Fig. 4B). However, both the 1.875 µM and 15 µM doses had substantial variation between replicate samples, possibly due to insufficient inhibition and/or substrate depletion. Consistent with this, examination of the OCR curves revealed that injection of 3.75 µM and 7.5 µM FCCP resulted in a sustained increase in OCR (Fig. 4C). In contrast, the 1.875 µM and 15 µM doses caused an initial increase in OCR that was not maintained over time (Fig. 4C), possibly due to incomplete uncoupling and substrate exhaustion, respectively (please see Supplemental Fig. 2). Similar optimization experiments carried out for antimycin A (Fig. 4D) and rotenone (Fig. 4E) demonstrated the dose–response relationship for these drugs. Based on the results from these optimization experiments, oligomycin (25 µg/mL), FCCP:pyruvate (7.5 µM:7.5 mM), and rotenone (10 µM) + antimycin (5 µM) were used to generate a OCR curve in cortical tissue punches (Fig. 4F) that is qualitatively similar to that obtained using isolated mitochondria (please see Fig. 1B for comparison).

### Respiration measurement in discrete anatomical regions

**Figure 5:**
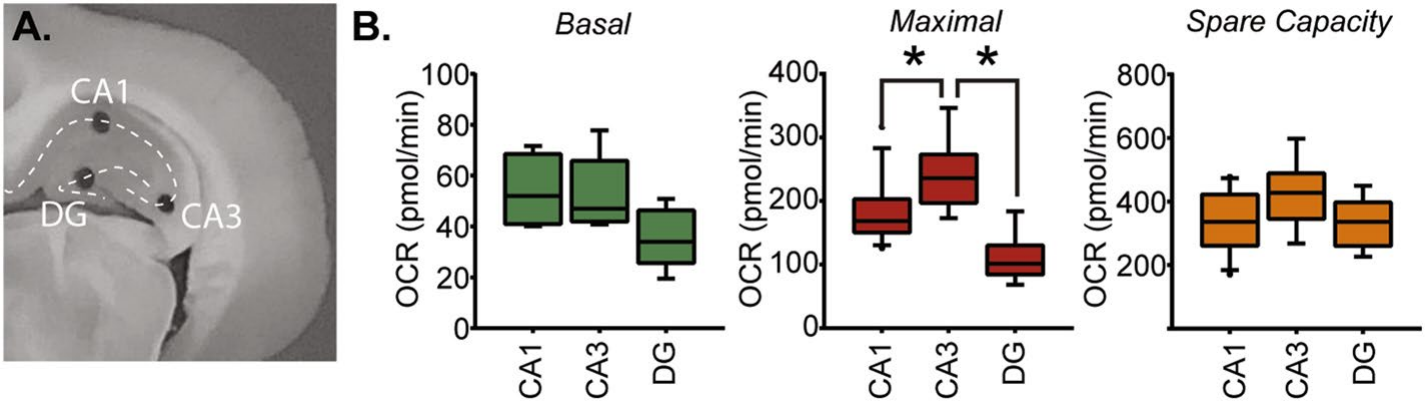
OCR measurements in punches taken from different hippocampal subfields. (**A**) A picture of a coronal rat brain section containing the dorsal hippocampus showing the location of the tissue punches (0.5 mm) used to assess OCR in the CA1, CA3, and dentate gyrus (DG) subfields. (**B**) Box and whisker plots showing the relative basal respiration, maximal respiration, and spare capacity between the CA1, CA3 and DG subfields. *, p < 0.05.

One of the key purposes for developing this method was to assess mitochondrial respiration within a specific brain structure/substructure, allowing for a more accurate map of the respiratory changes that occur in response to trauma or disease. We focused on the hippocampus as this structure has well-defined subfields, including the dentate gyrus (DG), the CA3 subfield, and the CA1 subfield (Fig. 5A). The electrophysiological properties of the neurons within each subfield have been well characterized, and their roles in learning and memory are established^42–47^. Furthermore, it has been demonstrated that individual hippocampal subfields are more vulnerable to specific insults (e.g. CA1 following ischemic stroke; CA3 following epileptic seizures)^48–50^. We applied our method to assess if respiration in these different subfields can be measured discreetly, and independently from each other. The summary results presented in Fig. 5B show that while CA1 and CA3 cell layer punches (0.5 mm in diameter) have similar basal OCR, the more densely packed dentate gyrus granule cell layer OCR is comparably lower. Interestingly, maximal respiration was found to be the highest in the CA3 subfield (F = 9.86, p = 0.0008). Spare capacity was comparable across all subfields, suggesting that under conditions of increased energetic demand, these regions should have a comparable capacity to increase ATP synthesis.

### Mild fluid percussion injury (mFPI) differentially alters mitochondrial respiration of hippocampal subfields

**Figure 6:**
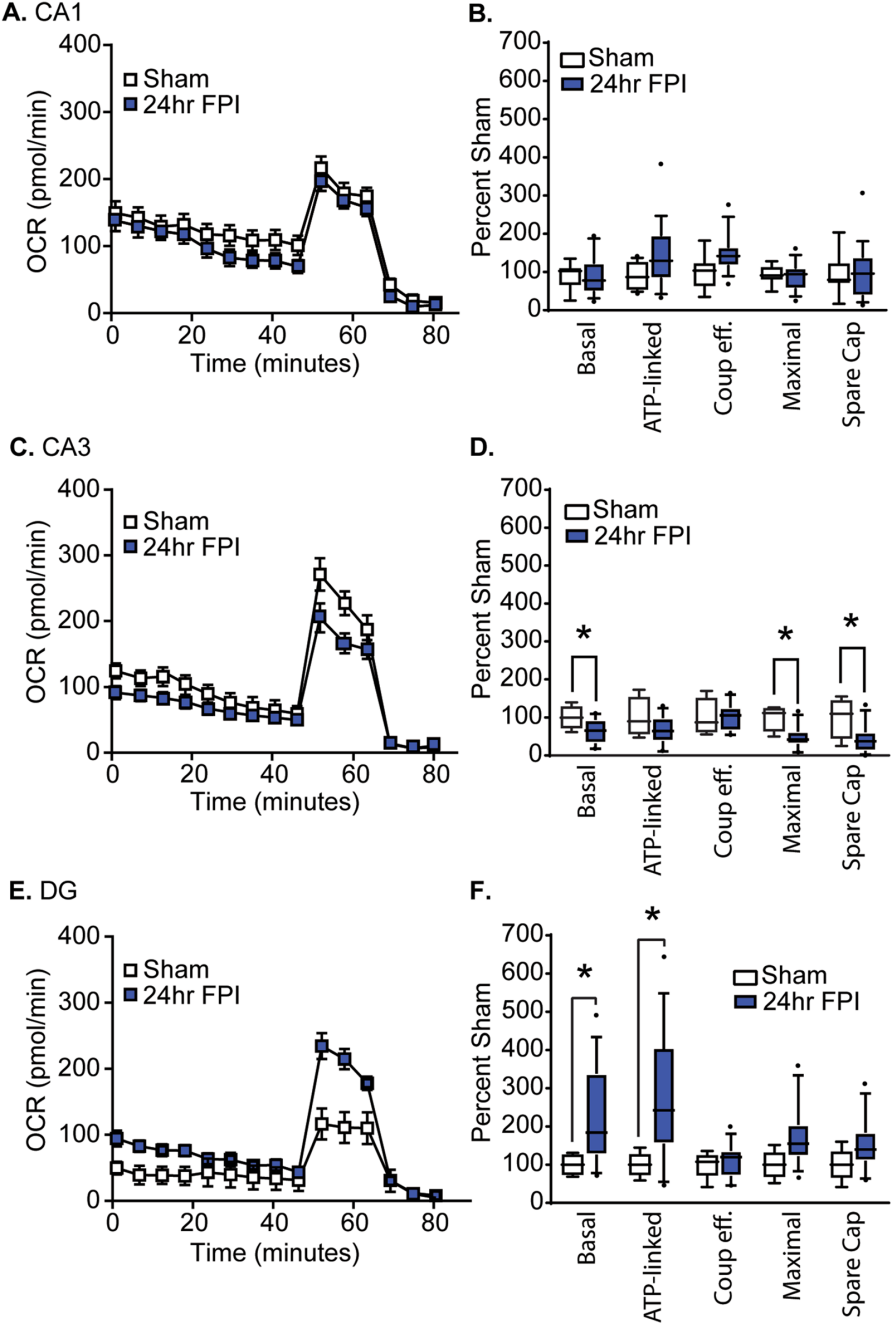
Mild fluid percussion injury (mFPI) differentially alters mitochondrial function across hippocampal subfields. Summary OCR data for punches excised from the (**A, B**) CA1, (**C, D**) CA3, and (**E, F**) dentate gyrus (DG) of sham (n = 4) and 24 h post-mFPI (n = 6) rats. Boxes indicate the 25th and 75th percentile. Whiskers represent the 5th and 95th percentile. Horizontal line represents the mean. *, significant differences between groups (*post-hoc* analysis after two-way ANOVA and correction for multiple comparisons).

To address the consequence of a mild traumatic brain injury (mTBI, often referred to as concussion) on respiration of hippocampal subfields, we measured OCR in tissue punches taken from the CA1, CA3, and dentate gyrus subfields 24 h after a single mFPI. From these measures, basal respiration, ATP-linked respiration, maximal respiration, spare capacity and coupling efficiency (the proportion of basal OCR used to drive ATP synthesis) were calculated and compared. A two-way ANOVA revealed no significant differences in CA1 mitochondrial respiration measures between sham and mFPI rats (F = 3.246, p = 0.074; Fig. 6A). In contrast, mFPI caused a significant suppression in respiration of CA3 tissue punches (*F* = 12.814, *p* < 0.001). This decrease was due to reductions in basal and maximal respiration, and a decrease in spare capacity (Fig. 6B). Interestingly, both basal and ATP-linked respiration were dramatically increased (*F* = 15.023, *p* < 0.001) in the dentate gyrus subfield after mFPI as compared to sham-operated controls (Fig. 6C).

### mFPI alters white matter respiration

**Figure 7:**
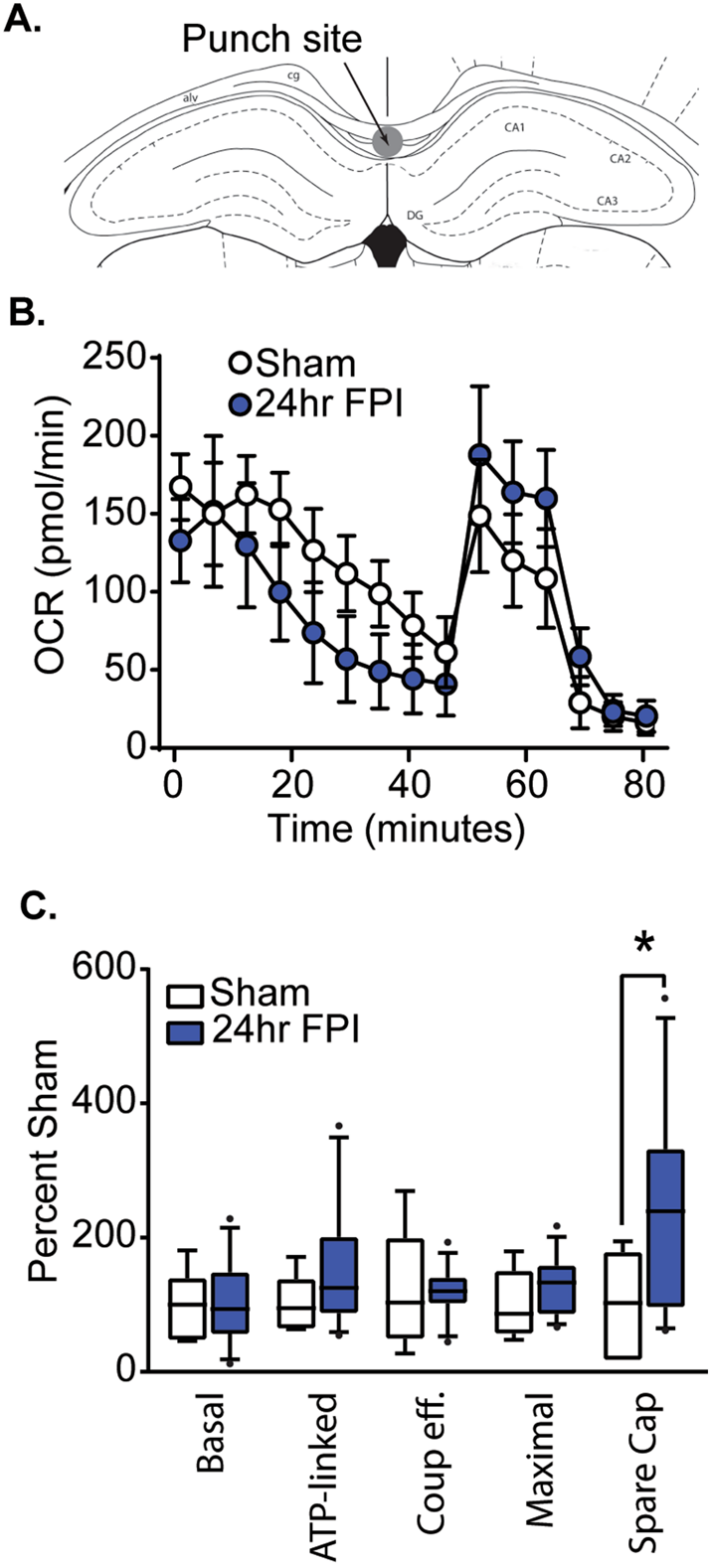
Measurements of OCR in punches taken from white matter tracts. (**A**) A drawing of a coronal rat brain section showing the position of the punch excised from the corpus callosum. (**B**) Representative OCR curves and (**C**) summary data for tissue punches taken from the corpus callosa of sham (n = 4) and mFPI (n = 6) rats. *, significant difference between groups (post-hoc analysis after two-way ANOVA and correction for multiple comparisons). Boxes are 25th and 75th percentile. Whiskers represent the 5th and 95th percentile. Horizontal line represents the mean.

White matter damage is routinely detected after TBI of all severity in humans and experimental animal models. We next examined if respiration of excised white matter can be measured using our new method, and if it is altered in response to a mFPI. Figure 7A shows a drawing of a coronal tissue section indicating the site from which white matter tissue punches were taken (these punches contain both the corpus callosum and hippocampal commissure). This area was chosen as mFPI has been shown to cause axonal damage within this region^51^. In preliminary experiments, we found that a 0.75 mm diameter tissue punch was required to generate basal OCR within the 20–200 pmol/min range, and the punch could still be constrained within the white matter tract without impinging on the surrounding gray matter. Figure 7B shows representative OCR curves from white matter punches obtained from sham (n = 4) and mFPI rats (n = 6). A two-way ANOVA revealed a significant difference between the two groups (*F* = 5.953, *p* = 0.017), with *post-hoc* analysis indicating that mFPI caused a significant increase in spare capacity within the corpus callosum (Fig. 7C).

## Discussion

Mitochondria play a critical role in meeting the energy requirements of the brain, and mitochondria dysfunction has been linked to the pathology of a number of brain diseases^7–19^. As different brain structures have different energy requirements, and may also have varying susceptibility to neurodegenerative diseases, it is of interest to measure mitochondrial function in anatomically defined regions. In this paper, we present a method that may be useful to researchers interested in examining the effect of brain diseases on mitochondria function by allowing them to directly measure mitochondrial respiration in spatially restricted brain regions without the need for isolation. The method we have developed uses a Seahorse XFe96 analyzer to measure mitochondrial respiration directly in small brain tissue punches without the necessity of first purifying mitochondria. This has the benefits of allowing respiration measurements from small, anatomically defined brain regions, limits possible mechanical damage incurred during purification, and retains the surrounding cellular milieu that may contain important factors impacting respiration. This method will allow researchers to map local changes in mitochondrial energetics in limited numbers of animals and at a spatial resolution not feasible with traditional mitochondria isolation approaches.

Utilizing well-established methods routinely used in slice physiology, we demonstrate that brain tissue slices can be maintained in oxygenated artificial CSF for at least 2 h prior to preparing biopsy punches without significant loss of respiratory activity. Once punches are prepared, they are incubated in 37 °C assay buffer for 30 min prior to performing respiration measurements. This approach of resting sections prior to incubation in assay buffer is consistent with methods used to obtain reproducible long-term potentiation (LTP) measurements in brain slices^52^. Employing this methodology, we show that tissue sections can be stored for up to 2 h before assaying without appreciable changes in basal activity over the subsequent 2 h monitoring period. Although we did not test longer storage times, brains sections used for electrophysiology have been reported to be stable for up to 6 h after preparation, and under certain conditions even longer, suggesting that prolonged storage of tissues intended for respiration measurements may be feasible^38^.

Using tissue punches from the parietal cortex, we determined that there is a proportional relationship between the punch size and OCR measurement, up to a punch diameter of 1.5 mm. In punches larger than 1.5 mm diameter, we observed a decrease in OCR. While the reason for this is not clear, it may be the result of substrate or oxygen depletion in the reaction buffer due to the increased volume of respiring tissue. Importantly, although basal respiration rate is increased in proportion to diameter/surface area, a higher basal respiration rate can compromise the instruments ability to measure key mitochondria functions, such as maximal respiration and spare capacity, due reduced responsivity to the uncoupler FCCP. For this reason, we recommend cortical respiration be measured using 0.5 mm diameter and 220 µm in thickness tissue punches. In the hippocampus, up to 0.75 mm diameter biopsies can be used. However, care must be taken to avoid inclusion of the overlying white matter or encroaching upon different hippocampal subfields when using punch diameters this large. Alternatively, customized biopsy needles with specialized shapes, such as an oval or rectangle, could be made to maximize the number of neuronal cell bodies captured within the punch while minimizing the amount of neighboring cells/white matter tracts.

Although we tested the relationship between punch diameter and respiration, we only examined biopsies excised from 220 µm thick brain sections. This thickness was chosen as the distance between the Seahorse XFe96 oxygen sensor probe and the plate is generally less than 1 mm, and suitable for a sample that is up to 300 μm thick^53,54^. A thicker tissue sample can be damaged by the probe during the assay, potentially altering mitochondrial and/or probe function and leading to variable results. However, punches can be excised from brain sections thinner than 220 μm as long as the basal OCR readings stay within 20–200 pmol/min. Adjusting the tissue section thickness may be one way to prepare sufficient numbers of biopsy punches/animal from smaller structures. In our studies, we have determined that 4 tissue biopsies are need for each brain region to reduce the coefficient of variation to ≤ 5%. This is consistent with data collected from organotypic hippocampal slices assayed using the Seahorse XFe24 analyzer, which found that a minimum of 4–5 tissue punches/location/animal were needed for replication^29,40^. Given this degree of replication, and utilizing one well per tissue punch (including 4 wells for blanks), our developed method allows for examination of up to 23 animals, brain regions, or experimental conditions in a single 96-well experiment. However, it is important to note that movement of the tissue punch from the center of the well during the assay procedure can cause artificially low OCR readings and may require the elimination of punches from the analysis. In our hands, < 5% of assayed punches were found to have moved during the assay, likely due to probe movement during injections and mixing. Although not specifically tested, it is plausible that a method of adhering the tissue punch to the bottom center of the well using gelatin or polyethylenimine can be used to minimize tissue movement during the assay procedure. However, the effect of these coatings on tissue respiration needs to be tested prior to their use.

Our drug optimization experiments revealed that the final concentrations of mitochondrial inhibitors in the reaction wells was higher than typically used in respiration assays for isolated mitochondria or cultured cells. We found that the degree of inhibition achieved using lower doses was sub-maximal, possibly due to limited penetration of these agents resulting in a lower local concentration within the core of the tissue punch. When higher concentrations were used, a stable effect was achieved within 20 min of drug application, allowing us to shorten the overall assay time to 2 h. However, it is important to note that use of oligomycin > 25 µg/ml or FCCP > 15 µM resulted in increased variance across replicates. Although not specifically tested in this method, oligomycin at high concentrations can cause spontaneous uncoupling via excessive proton leak^55^, an effect that might have contributed to the increased variance we observed at the 25 µg/ml dose. Likewise, high concentrations of FCCP may have resulted in substrate exhaustion and failure to maintain a stable elevation in OCR. If an experiment requires using a higher concentration of FCCP, inclusion of additional substrate (e.g. pyruvate) with the uncoupler injection can be used to reduce variance (Supplemental Fig. 2) and accurately measure maximal OCR. Finally, while the drug doses described herein were optimized for rat hippocampal and cortical tissue punches, doses should be empirically tested in other structures (or other species) to avoid complications related to incomplete inhibition, substrate exhaustion, and/or spontaneous uncoupling.

While cells in the CA1, CA3, and dentate gyrus hippocampal subfields have been shown to respond differently to TBI dependent on injury severity^56^, to our knowledge, the function of mitochondria isolated from these discrete brain regions have not been assessed following injury. As isolating mitochondria from a tissue biopsy would be methodologically difficult, one advantage of the current method is that it enables the user to measure mitochondrial respiration in spatially restricted brain regions/subregions. Using this method, we find that the granule neurons of the dentate gyrus have relatively lower basal respiration, despite their higher density, by comparison to the pyramidal neurons in the CA1 and CA3 subfield. It has been reported that spatial information is sparsely represented by granule neurons (i.e. a minority of neurons are highly active and dominates information coding) which enables dentate gyrus to carry out pattern separation^57–59^. Thus, the lower basal respiration we observed may be reflective of firing of a small percent of granule neurons. In addition, we observed that the maximal OCR in the CA3 subfield was significantly higher than that detected in the CA1 subfield, despite having similar basal respiration. The CA3 subfield has recurrent collateral projections that are thought to be important for a variety of cognitive functions including pattern completion and spatial working memory^60,61^. It is possible that the increased maximal respiration of CA3 neurons may be required for maintenance of sustained activity that is necessary for working memory and pattern completion.

Our results using biopsy punches taken from the different subfields of the hippocampus revealed that mFPI differentially alters mitochondrial respiration within these subfields (Fig. 6). Specifically, we observed that the CA3 subfield in injured rats had decreased basal and maximal respiration, whereas the dentate gyrus had significantly increased basal and ATP-linked respiration. While the reason for these changes are not clear at present, they may be related to mitochondrial size^62^, cristae density^63^, alterations in the levels/activity of respiratory complex proteins^64–66^, or substrate availability^67^. For example, although pyruvate is added to the reaction mixture during assessment of maximal respiration, it must be transported into the cells using monocarboxylate transporters (MCTs) and into mitochondria by voltage-dependent anion channels (VDACs) and mitochondrial pyruvate carriers (MPCs). A decrease in the levels of these transporters could give rise to the reduced respiration we observed in the CA3 subfield. Recently, it has been demonstrated that a reduction in the activity of 2-oxoglutarate dehydrogenase complex (OGDHC) is associated with reduced mitochondrial function in the cortex after TBI^68^. Although not yet examined in the hippocampus, as OGDHC is a key regulatory step in the production of NADH, a reduction in its activity could give rise to the decrease in CA3 respiration we observed. Regardless of the mechanism, as CA3 neurons are particularly vulnerable to TBI^69–71^, this decrease in metabolism observed acutely after injury may contribute to the ultimate demise of these cells. Alternatively, a decrease in respiration may represent a protective mechanism in an attempt to reduce free radical production. Future studies would be required to distinguish between these possibilities.

White matter loss is also often observed in neurologic diseases such as multiple sclerosis and neurodegenerative aging^72–74^. Furthermore, a large body of clinical and experimental data has shown that white matter damage is a consistent pathology of mild, moderate and severe TBI^41,75–78^, and can be observed in the absence of overt gray matter damage^76,79–81^. However, due to the relatively large amount of tissue required to isolate mitochondria, it has not been examined if mitochondrial respiration is altered in damaged white matter tracts after TBI. Previous PET and autoradiograph studies have shown that gray matter consumes more glucose (thus, more blood flows to gray matter) as compared to white matter. In agreement with these measurements, our respiration measurement of excised corpus callosum (including the underlying hippocampal commissure) indicated that larger tissue punches (0.75 mm for white matter compared to 0.5 mm for gray matter) were required to measure mitochondrial respiration in this white matter tract. When examined after mild FPI, we observed a significant increase in spare respiratory capacity. Spare capacity, the difference between basal and maximal respiration, is considered to be an important facet of mitochondrial function. When cells are subjected to stress, energy demand increases, requiring more ATP to maintain cellular function. Thus, the increase in spare capacity we observed may indicate a compensatory mechanism to allow more ATP production to overcome stress. Alternatively, this change may be reflective of astrocyte proliferation and/or infiltration of inflammatory cells that has been previously observed to occur in white matter tracts after TBI^51,82,83^.

Although the method we have developed has several advantages over conventional mitochondrial isolation measures, this method does have weaknesses. First, mechanical damage to the brain tissue during sectioning and biopsy may alter tissue respiration. Second, the mixed population of cells present in the tissue punch is not amenable to identifying the cell types that have altered mitochondrial respiration. Third, in brains with ongoing pathology, immune cells (both resident and circulating) can infiltrate the tissue and contribute to the respiration measurements, complicating interpretation. Fourth, although we demonstrate that this method can be used to assess mitochondrial respiration in white matter tracts, the basal respiration rate in these fiber tracts is low. This low basal OCR may present a basement effect problem and compromise the ability to accurately assess the degree of inhibition when using agents such as oligomycin that are expected to reduce OCR. Although larger tissue punches up to about 1 mm diameter can be used to increase the basal respiration, we found that white matter tissue biopsies tended to float in the reaction wells, presumably due to their high lipid content. Fifth, in some situations, it may be necessary to use an external normalization factor across different tissues. This may be especially important in areas that suffer from cell loss or infiltration of cells. Complementary western blot analysis using cell specific markers may be required to assess these changes. Although the addition of BSA to the assay medium has been reported to enhance the stability of mitochondria and be necessary for achieving maximal respiration with FCCP injection^40^, this can compromise the use of the tissue for subsequent assays (e.g. for assaying protein concentrations). However, we found that rested tissues can be assayed without BSA. However, the variability of OCR is larger and further optimization of drug concentrations will be required (Supplemental Fig. 3). Finally, the preset method was optimized for examining mitochondrial respiration. Although the Seahorse XFe96 analyzer also monitors the extracellular acidification rate (ECAR), the use of this data to garner information concerning glycolysis (e.g. glycolytic capacity, glycolytic reserve) would be erroneous as different manipulations (e.g. addition of glucose, 2-deoxyglucose) are required to accurately assess these functions. However, if developed, the combination of assessing mitochondrial function and glycolytic function in companion tissue punches would be advantageous over approaches based on isolated mitochondria.

## Methods

### Animals

All experimental procedures were conducted in accordance with the Guide for the Care and Use of Laboratory Animals of the National Institutes of Health and were approved by the Institutional Animal Care and Use Committee (IACUC). Male Sprague–Dawley rats (275–300 g) were purchased from Envigo (Houston, Texas). Rats were group housed on a 12-h light/dark cycle, with ad libitum access to food and water. All experiments were performed during the light cycle.

### Preparation of brain slices

Rats were euthanized by decapitation using a sharp guillotine. Brains were rapidly removed (within 30 s of decapitation) and immersed in ice-cold (4–5 °C) artificial cerebrospinal fluid (aCSF; 120 mM NaCl, 3.5 mM KCl, 1.3 mM CaCl_2_, 1 mM MgCl_2_, 0.4 mM KH_2_PO_4_, 5 mM HEPES, and 10 mM D-glucose; pH 7.4) that had been oxygenated for 1 h using 95% O_2_:5% CO_2_. Coronal Sections (220 μm) were prepared using a modified McIlwain tissue chopper (Ted Pella. Inc.; Redding, CA) with a chilled stage and blade, then transferred to a holding chamber containing continuously oxygenated aCSF at room temperature (~ 23 °C).

### Tissue punches and respiration measurements

Brain sections were individually transferred to a biopsy chamber containing fresh oxygenated aCSF. Stainless steel WellTech Rapid-Core biopsy punch needles (350 µm, 500 µm, 750 µm, 1.0 mm, 1.5 mm, 2.0 mm, or 3.0 mm diameter; World Precision Instruments; Sarasota, FL) were used to excise the tissue punches. In uninjured rats, tissue punches were taken from both hemispheres for each anatomical location using three consecutive coronal sections (i.e. a total of 6 punches for each anatomical structure). Punches were ejected directly into an XFe96 Cell Culture Microplate (101085-004; Agilent Technologies, Santa Rosa, CA) based on a pre-determined plate layout. Each well contained 180 µL room temperature assay media (aCSF supplemented with 0.6 mM pyruvate and 4 mg/ml BSA). After loading all biopsy samples, each well was visually inspected to ensure that the punches were submerged and centered at the bottom of the well. The XFe96 Cell Culture Microplate was then incubated at 37 °C for approximately 30 min.

During this incubation period, 10 × concentration of assay drugs (prepared in aCSF) were loaded into their respective injection ports of a hydrated (overnight in distilled water, exchanged for XF Calibrant solution 3 h prior to assay initiation) Seahorse XFe96 Extracellular Flux Assay sensor cartridge. The sensor cartridge containing the study drugs was then inserted into the analyzer for calibration. Once the analyzer was calibrated, the calibration plate was replaced by the microplate containing the tissue punches and the assay protocol initiated.

### Mitochondria isolation and respiration measurements

Mitochondria were isolated using Percoll density gradient centrifugation as previously described^84,85^. Briefly, cortical tissue from a male Sprague Dawley rat was homogenized in ice-cold isolation buffer (100 mM Tris pH 7.4, 10 mM EDTA, 12% Percoll solution, 1 mM sodium fluoride, 1 mM sodium molybdate, 100 nM okadaic acid, 1 mM PMSF and 10 µg/ml leupeptin) using a Dounce homogenizer. Tissue was homogenized using 4 strokes with the loose pestle (clearance 72–120 µm), followed by 8 strokes using the tight pestle (clearance 20–56 µm). The homogenate was then layered onto a discontinuous Percoll gradient (26% and 40% Percoll) and centrifuged for 10 min (30,700* g* at 4 °C). The enriched mitochondrial fraction was removed from the 26–40% interface, transferred to individual centrifuge tubes, and washed with isolation buffer. Mitochondrial fractions were pelletized by centrifugation (16,700* g* at 4 °C) for 10 min, briefly washed in isolation buffer to remove the Percoll, then re-centrifuged (16,700 *g* at 4 °C for 10 min) and used for respiration assays. The basic respiration assay buffer (mitochondrial assay solution; MAS) contains 70 mM sucrose, 220 mM mannitol, 10 mM KH_2_PO_4_, 5 mM MgCl_2_, 2 mM HEPES, and 1 mM EGTA. For carrying out respiration using isolated mitochondria, pyruvate (10 mM) and malate (5 mM) were added to the MAS, and the resulting solution used to make 10 × stocks of the respiratory inhibitors and uncouplers. The respiratory stocks were loaded into the drug ports of a hydrated sensor cartridge in the following order: (A) oligomycin (2.5 µg/mL final), (B) FCCP (4 µM final), and (C) antimycin A (4 µM final) + rotenone (2 µM final). The protein concentrations of isolated mitochondria preparations were measured. Equal amounts of mitochondria (1 µg protein/well) were plated on the Seahorse cell culture microplate in 20 µL of MAS + substrate + 0.2% w/v fatty-acid free BSA and centrifuged at 2,000 × *g* for 20 min at 4 °C. The assay medium (MAS + substrates + 0.2% BSA + 4.5 mM ADP) was then added to the wells to bring the final volume to 180 µL prior to the plate being incubated at 37 °C for 30 min and transferred to the analyzer for analysis.

The tissue respiration assay protocol consisted of a minimum of three cycles of OCR measurements for each measurement period. Each cycle consisted of a 2 min ‘mix’ period and 2 min ‘wait’ period, followed by a 3 min ‘measure’ period. For tissue punch diameter and drug concentration optimization, the number of cycles varied based on the phase being optimized. This was done in order to achieve stable recordings at maximal drug effect without compromising the viability of the tissue. For measuring OCR of tissue punches excised from coronal sections of mFPI brains, slight modifications were made to the above protocol. Three cycles were used to obtain a basal OCR, 6 cycles were used to assess the effect of the F1-Fo ATP synthase inhibitor oligomycin, three cycles were used to evaluate the effect of the uncoupler FCCP, and 3 cycles were used to measure mitochondria-associated respiration following injection of antimycin A/rotenone.

### Lateral mild fluid percussion injury (mFPI)

Lateral mFPI was carried out essentially as described previously^51,86–88^. Briefly, rats were anesthetized using 5% isoflurane with a 1:1 N_2_O/O_2_ mixture and then maintained with a 2.5% isoflurane with 1:1 air/O_2_ mixture via a face mask. Animals were mounted on a stereotaxic frame, a midline 4.8 mm diameter craniectomy was made midway between bregma and lambda. A hub (modified from a 20-gauge needle) for the delivery of the fluid pulse was fitted into the burr hole and held in place using cyanoacrylate and dental cement. The rat was then injured using a FPI device at a pressure of 1.5 atmosphere (atm) above room pressure. Immediately after injury, the hub and surrounding dental cement were removed and the incision closed by wound clips. Sham-operated animals received all the aforementioned surgical procedures except hub implantation and the injury. The animals’ body temperature was maintained at 37 °C during the surgery using a rectal thermometer coupled to a heating pad. Brain tissues from sham and injured brains were collected for respiration assays 24 h after the injury or sham surgery.

### Data analysis

Upon completion of the assay, the OCR curve generated by each well was examined using Agilent’s Wave 2.6.0 software. The XFe96 training manual recommends that the optimum signal range for basal OCR should be between 20–160 pmol/min. Wells with low basal activity (< 20 pmol/min) were excluded from analysis. Similarly, samples that failed to respond to FCCP/pyruvate were also excluded, as this represents a complete failure of the assay, possibly due to movement of the tissue punch during mixing cycles. In a typical experiment, 5% or fewer wells were excluded. Normalization across plates was achieved by converting all metabolic output calculations to a percent change from the control baseline samples present in each assay.

## Supplementary information

Supplementary information

## Data Availability

All data generated or analyzed during this study are included in this published article (and its Supplementary Information files).

## Abbreviations

aCSF: Artificial cerebrospinal fluid
FCCP: Carbonyl cyanide-4 (trifluoromethoxy) phenylhydrazone
mFPI: Mild fluid percussion injury
MAS: Mitochondrial assay solution
mTBI: Mild traumatic brain injury
OCR: Oxygen consumption rate

## References

[1] Funahashi M, Kohda H, Hori O, Hayashida H, Kimura H (1990). Potentiating effect of morphine upon d-methamphetamine-induced hyperthermia in mice. Effects of naloxone and haloperidol. Pharmacol. Biochem. Behav..

[2] Yu SB, Pekkurnaz G (2018). Mechanisms orchestrating mitochondrial dynamics for energy homeostasis. J. Mol. Biol..

[3] Flippo KH, Strack S (2017). Mitochondrial dynamics in neuronal injury, development and plasticity. J. Cell Sci..

[4] Graier WF, Frieden M, Malli R (2007). Mitochondria and Ca(2+) signaling: Old guests, new functions. Pflugers Arch..

[5] Scherz-Shouval R, Elazar Z (2011). Regulation of autophagy by ROS: Physiology and pathology. Trends Biochem. Sci..

[6] Dasgupta S (2019). Mitochondrion: I am more than a fuel server. Ann. Transl. Med..

[7] Nunnari J, Suomalainen A (2012). Mitochondria: In sickness and in health. Cell.

[8] Cho DH, Nakamura T, Lipton SA (2010). Mitochondrial dynamics in cell death and neurodegeneration. Cell Mol. Life Sci..

[9] Yonutas HM, Vekaria HJ, Sullivan PG (2016). Mitochondrial specific therapeutic targets following brain injury. Brain Res..

[10] Gajavelli S (2015). Evidence to support mitochondrial neuroprotection, in severe traumatic brain injury. J. Bioenerg. Biomembr..

[11] Mazzeo AT, Beat A, Singh A, Bullock MR (2009). The role of mitochondrial transition pore, and its modulation, in traumatic brain injury and delayed neurodegeneration after TBI. Exp. Neurol..

[12] Singh IN, Sullivan PG, Deng Y, Mbye LH, Hall ED (2006). Time course of post-traumatic mitochondrial oxidative damage and dysfunction in a mouse model of focal traumatic brain injury: Implications for neuroprotective therapy. J. Cereb. Blood Flow Metab.

[13] Narendra DP, Youle RJ (2012). Neurodegeneration: Trouble in the cell's powerhouse. Nature.

[14] Lin MT, Beal MF (2006). Mitochondrial dysfunction and oxidative stress in neurodegenerative diseases. Nature.

[15] Alexiou A (2018). Mitochondrial dynamics and proteins related to neurodegenerative diseases. Curr. Protein Pept. Sci..

[16] Cai Q, Tammineni P (2017). Mitochondrial aspects of synaptic dysfunction in Alzheimer's disease. J. Alzheimers. Dis..

[17] Grimm A, Eckert A (2017). Brain aging and neurodegeneration: From a mitochondrial point of view. J. Neurochem..

[18] Panchal K, Tiwari AK (2019). Mitochondrial dynamics, a key executioner in neurodegenerative diseases. Mitochondrion.

[19] Pandya JD (2016). Advanced and high-throughput method for mitochondrial bioenergetics evaluation in neurotrauma. Methods Mol. Biol..

[20] Braak H, Braak E (1996). Evolution of the neuropathology of Alzheimer's disease. Acta Neurol. Scand. Suppl..

[21] Devenney E (2015). Progression in behavioral variant frontotemporal dementia: A longitudinal study. JAMA Neurol..

[22] Matsuda H (2016). MRI morphometry in Alzheimer's disease. Ageing Res. Rev..

[23] Aisen PS (2017). On the path to 2025: understanding the Alzheimer's disease continuum. Alzheimers Res. Ther..

[24] Veitch DP (2019). Understanding disease progression and improving Alzheimer's disease clinical trials: Recent highlights from the Alzheimer's disease neuroimaging initiative. Alzheimers Dement..

[25] Dubois B (2016). Preclinical Alzheimer's disease: Definition, natural history, and diagnostic criteria. Alzheimers Dement..

[26] Picard M (2011). Mitochondrial structure and function are disrupted by standard isolation methods. PLoS ONE.

[27] Picard M (2010). Mitochondrial functional impairment with aging is exaggerated in isolated mitochondria compared to permeabilized myofibers. Aging Cell.

[28] Picard M, Taivassalo T, Gouspillou G, Hepple RT (2011). Mitochondria: Isolation, structure and function. J Physiol.

[29] Fried NT, Moffat C, Seifert EL, Oshinsky ML (2014). Functional mitochondrial analysis in acute brain sections from adult rats reveals mitochondrial dysfunction in a rat model of migraine. Am. J. Physiol. Cell Physiol..

[30] Schniertshauer D, Gebhard D, Bergemann J (2018). Age-dependent loss of mitochondrial function in epithelial tissue can be reversed by coenzyme Q10. J. Aging Res..

[31] Neville KE (2018). A novel ex vivo method for measuring whole brain metabolism in model systems. J. Neurosci. Methods.

[32] Shintaku J, Guttridge DC (2016). Analysis of aerobic respiration in intact skeletal muscle tissue by microplate-based respirometry. Methods Mol. Biol..

[33] Bugge A, Dib L, Collins S (2014). Measuring respiratory activity of adipocytes and adipose tissues in real time. Methods Enzymol.

[34] Leung DTH, Chu S (2018). Measurement of oxidative stress: Mitochondrial function using the seahorse system. Methods Mol. Biol..

[35] Iuso A, Repp B, Biagosch C, Terrile C, Prokisch H (2017). Assessing mitochondrial bioenergetics in isolated mitochondria from various mouse tissues using seahorse XF96 Analyzer. Methods Mol. Biol..

[36] Sperling JA (2019). Measuring respiration in isolated murine brain mitochondria: Implications for mechanistic stroke studies. Neuromol. Med..

[37] Dondzillo A (2015). A recording chamber for small volume slice electrophysiology. J. Neurophysiol..

[38] Cameron MA (2017). Prolonged incubation of acute neuronal tissue for electrophysiology and calcium-imaging. J. Vis. Exp..

[39] O'Halloran KD (2016). Blast from the past! Phrenic motor memory of antecedent episodic hypercapnia is serotonin dependent: Relevance to respiratory rehabilitation and sleep-disordered breathing?. Exp. Physiol..

[40] Schuh RA (2011). Adaptation of microplate-based respirometry for hippocampal slices and analysis of respiratory capacity. J. Neurosci. Res..

[41] Maynard ME (2019). Carnosic acid improves outcome after repetitive mild traumatic brain injury. J. Neurotrauma..

[42] Tuncdemir SN, Lacefield CO, Hen R (2019). Contributions of adult neurogenesis to dentate gyrus network activity and computations. Behav. Brain Res..

[43] Kesner RP (2018). An analysis of dentate gyrus function (an update). Behav. Brain Res..

[44] Knierim JJ, Neunuebel JP (2016). Tracking the flow of hippocampal computation: Pattern separation, pattern completion, and attractor dynamics. Neurobiol. Learn. Mem..

[45] Rebola N, Carta M, Mulle C (2017). Operation and plasticity of hippocampal CA3 circuits: Implications for memory encoding. Nat. Rev. Neurosci..

[46] Kesner RP (2007). Behavioral functions of the CA3 subregion of the hippocampus. Learn. Mem..

[47] Kesner RP, Lee I, Gilbert P (2004). A behavioral assessment of hippocampal function based on a subregional analysis. Rev. Neurosci..

[48] Bartsch T (2015). Selective neuronal vulnerability of human hippocampal CA1 neurons: Lesion evolution, temporal course, and pattern of hippocampal damage in diffusion-weighted MR imaging. J. Cereb. Blood Flow Metab..

[49] Medvedeva YV, Ji SG, Yin HZ, Weiss JH (2017). Differential vulnerability of CA1 versus CA3 pyramidal neurons after ischemia: Possible relationship to sources of Zn2+ accumulation and its entry into and prolonged effects on mitochondria. J. Neurosci..

[50] Song H (2018). Contributions of the hippocampal CA3 circuitry to acute seizures and hyperexcitability responses in mouse models of brain ischemia. Front. Cell Neurosci..

[51] Hylin MJ (2013). Behavioral and histopathological alterations resulting from mild fluid percussion injury. J. Neurotrauma.

[52] Papouin T, Haydon PG (2018). Obtaining acute brain slices. Bio Protoc.

[53] 53Ferrick, A. N. T. W. Analysis of metabolic activity in cells using extracellular flux rate measurements. *United States patent* US20070087401A1 (2007).

[54] Orrell, J. A. N. S. Y. J. *United States patent* US 20160077083 A1 (2014)

[55] Jastroch M, Divakaruni AS, Mookerjee S, Treberg JR, Brand MD (2010). Mitochondrial proton and electron leaks. Essays Biochem..

[56] Saatman KE, Feeko KJ, Pape RL, Raghupathi R (2006). Differential behavioral and histopathological responses to graded cortical impact injury in mice. J. Neurotrauma.

[57] GoodSmith D (2017). Spatial representations of granule cells and mossy cells of the dentate gyrus. Neuron.

[58] Schmidt B, Marrone DF, Markus EJ (2012). Disambiguating the similar: The dentate gyrus and pattern separation. Behav. Brain Res..

[59] Neunuebel JP, Knierim JJ (2014). CA3 retrieves coherent representations from degraded input: Direct evidence for CA3 pattern completion and dentate gyrus pattern separation. Neuron.

[60] Gilbert PE, Brushfield AM (2009). The role of the CA3 hippocampal subregion in spatial memory: A process oriented behavioral assessment. Prog. Neuropsychopharmacol. Biol. Psychiatry.

[61] Le Duigou C, Simonnet J, Telenczuk MT, Fricker D, Miles R (2014). Recurrent synapses and circuits in the CA3 region of the hippocampus: An associative network. Front. Cell Neurosci..

[62] Fischer TD (2016). Altered mitochondrial dynamics and TBI pathophysiology. Front. Syst. Neurosci..

[63] Nielsen J (2017). Plasticity in mitochondrial cristae density allows metabolic capacity modulation in human skeletal muscle. J. Physiol..

[64] Pandya JD (2019). Comprehensive profile of acute mitochondrial dysfunction in a preclinical model of severe penetrating TBI. Front. Neurol..

[65] Kilbaugh TJ (2015). Mitochondrial bioenergetic alterations after focal traumatic brain injury in the immature brain. Exp. Neurol..

[66] Chen H (2016). Moderate traumatic brain injury is linked to acute behaviour deficits and long term mitochondrial alterations. Clin. Exp. Pharmacol. Physiol..

[67] Xing G, Ren M, Watson WD, O'Neill JT, Verma A (2009). Traumatic brain injury-induced expression and phosphorylation of pyruvate dehydrogenase: A mechanism of dysregulated glucose metabolism. Neurosci. Lett..

[68] Mkrtchyan GV (1859). Thiamine preserves mitochondrial function in a rat model of traumatic brain injury, preventing inactivation of the 2-oxoglutarate dehydrogenase complex. Biochim. Biophys. Acta Bioenerg..

[69] Mao H, Elkin BS, Genthikatti VV, Morrison B, Yang KH (2013). Why is CA3 more vulnerable than CA1 in experimental models of controlled cortical impact-induced brain injury?. J. Neurotrauma.

[70] Anderson KJ, Miller KM, Fugaccia I, Scheff SW (2005). Regional distribution of fluoro-jade B staining in the hippocampus following traumatic brain injury. Exp. Neurol..

[71] Zanier ER, Lee SM, Vespa PM, Giza CC, Hovda DA (2003). Increased hippocampal CA3 vulnerability to low-level kainic acid following lateral fluid percussion injury. J. Neurotrauma.

[72] Hase Y, Horsburgh K, Ihara M, Kalaria RN (2018). White matter degeneration in vascular and other ageing-related dementias. J. Neurochem..

[73] Hinman JD, Abraham CR (2007). What's behind the decline? The role of white matter in brain aging. Neurochem. Res..

[74] Parr J (1975). The neuropathology of diseases of the white matter. Ann. Clin. Lab. Sci..

[75] Gold EM (2018). Repeated mild closed head injuries induce long-term white matter pathology and neuronal loss that are correlated with behavioral deficits. ASN Neuro.

[76] Johnson VE, Stewart W, Smith DH (2013). Axonal pathology in traumatic brain injury. Exp. Neurol..

[77] Wilde EA (2019). Persistent disruption of brain connectivity after sports-related concussion in a female athlete. J. Neurotrauma.

[78] Ware AL (2018). A preliminary investigation of corpus callosum subregion white matter vulnerability and relation to chronic outcome in boxers. Brain Imaging Behav..

[79] McGinn MJ (2009). Biochemical, structural, and biomarker evidence for calpain-mediated cytoskeletal change after diffuse brain injury uncomplicated by contusion. J. Neuropathol. Exp. Neurol.

[80] Yamamoto S, Levin HS, Prough DS (2018). Mild, moderate and severe: terminology implications for clinical and experimental traumatic brain injury. Curr. Opin. Neurol..

[81] Bramlett HM, Dietrich WD (2007). Progressive damage after brain and spinal cord injury: Pathomechanisms and treatment strategies. Prog. Brain Res..

[82] Burda JE, Bernstein AM, Sofroniew MV (2016). Astrocyte roles in traumatic brain injury. Exp Neurol.

[83] Adelson PD (2001). Histopathologic response of the immature rat to diffuse traumatic brain injury. J. Neurotrauma.

[84] Sims NR, Anderson MF (2008). Isolation of mitochondria from rat brain using Percoll density gradient centrifugation. Nat. Protoc.

[85] Fischer TD, Dash PK, Liu J, Waxham MN (2018). Morphology of mitochondria in spatially restricted axons revealed by cryo-electron tomography. PLoS Biol.

[86] Dixon CE (1987). A fluid percussion model of experimental brain injury in the rat. J. Neurosurg..

[87] Floyd CL, Golden KM, Black RT, Hamm RJ, Lyeth BG (2002). Craniectomy position affects morris water maze performance and hippocampal cell loss after parasagittal fluid percussion. J. Neurotrauma.

[88] Kelley BJ, Farkas O, Lifshitz J, Povlishock JT (2006). Traumatic axonal injury in the perisomatic domain triggers ultrarapid secondary axotomy and Wallerian degeneration. Exp. Neurol.

