# Supplementary material for: A method for assessing tissue respiration in anatomically defined brain regions
Source: Sci Rep. 2020 Aug 6;10:13179. doi: 10.1038/s41598-020-69867-2 (PMC7413397; doi:10.1038/s41598-020-69867-2)
Supplement: Supplementary file 1 — Supplementary information [file 41598_2020_69867_MOESM1_ESM.pdf]

A method for assessing tissue respiration in anatomically defined brain regions

Erica Underwood, John B. Redell, Jing Zhao, Anthony N. Moore, and Pramod K. Dash*
Department of Neurobiology and Anatomy, The University of Texas McGovern Medical School, Houston, Texas 77225

* To whom correspondence should be addressed:
P.K. Dash
Department of Neurobiology and Anatomy
The University of Texas Health Science Center at Houston (UTHealth)
P.O. Box 20708
Houston, TX 77225
Phone: (713) 500-5575, Fax: (713) 500-0621,
Email: p.dash@uth.tmc.edu

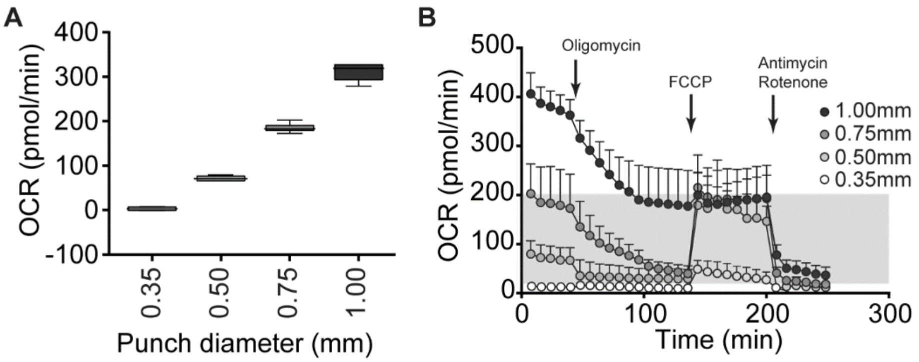

Supplemental Figure 1. Relationship between basal respiration and hippocampal punch diameter. To determine the optimal punch size for examining respiration in the rat hippocampus, punches of increasing diameter were prepared centered on the CA1 subfield. **A)** Box and whisker plots showing the basal OCR for the various hippocampal punch sizes. **B)** OCR curves showing the response of various punch diameters to mitochondrial inhibitors/uncoupler. Recommended range for OCR is indicated by the shaded area. Data are presented as mean ± SEM. Although increased basal respiration can be obtained in larger punch diameters, hippocampal punches ≥1 mm could not be accurately assayed due to ceiling effects as indicated by little to no response to FCCP. Punch sizes of 350 µm in diameter were too small and had basal respiration below the 20 pmol/min threshold.

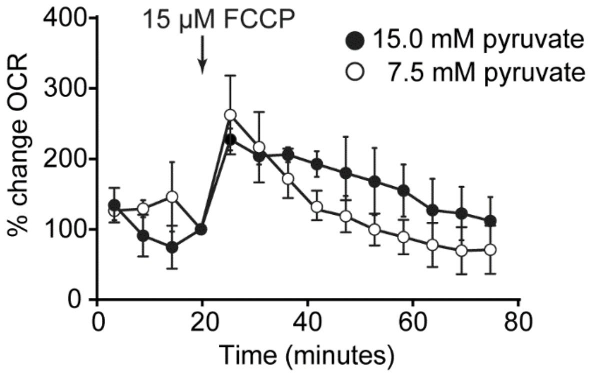

Supplemental Figure 2. High dose FCCP can cause substrate exhaustion. Representative OCR curves (generated using 0.5 mm diameter cortical tissue punches from a rat) demonstrating the response to 15 µM FCCP co-injected with either 7.5 mM or 15 mM pyruvate. Including 15 mM pyruvate in the reaction resulted a short (~15 min) plateau in elevated oxygen consumption after FCCP injection, though it was not stable over an extended time period.

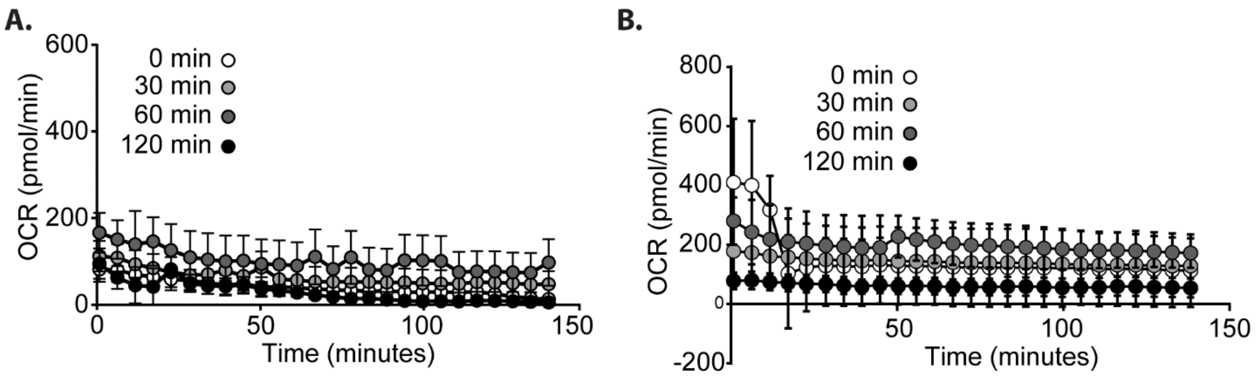

Supplemental Figure 3. Respiration can be measured without BSA in the assay buffer. Although the addition of BSA to the assay medium has been reported to enhance mitochondria stability, this can compromise subsequent manipulations (e.g. assaying protein concentration). We therefore tested tissue respiration in cortical tissue punches (after tissues were rested for various lengths of time in artificial CSF) when punches were assayed in the **A)** presence and **B)** absence of BSA. In the absence of BSA, brain sections rested for > 30 min prior to preparing tissue punches had relatively stable basal respiration, although punch-to-punch variability was higher. Punches that were assayed immediately after excision were found to have dramatically higher initial oxygen consumption in the absence of BSA, an effect not seen in tissue punches assayed in the presence of BSA. This suggests that if BSA needs to be eliminated from the assay mixture, a planned delay should be used to achieve a stable baseline.
